# Acid-modified clinoptilolite as a support for palladium–copper complexes catalyzing carbon monoxide oxidation with air oxygen

**DOI:** 10.1186/s13065-017-0256-6

**Published:** 2017-03-27

**Authors:** Tatyana L. Rakitskaya, Tatyana A. Kiose, Kristina O. Golubchik, Alim A. Ennan, Vitalia Y. Volkova

**Affiliations:** 1 Department of Inorganic Chemistry and Chemical Ecology, Odessa I.I. Mechnikov National University, 2, Dvoryanskaya St., Odessa, 65082 Ukraine; 2 Physicochemical Institute of Environment and Human Protection, 3, Preobrazhenskaya St., Odessa, 65082 Ukraine

**Keywords:** Clinoptilolite, Acid modification, FT-IR spectroscopy, XRD method, Water vapor adsorption, DTG/DTA, Palladium–copper catalysts, CO oxidation

## Abstract

Samples of natural clinoptilolite were modified by an acid–thermal method at nitric acid concentrations of 0.25, 0.5, 1.0, and 3.0 M and a contact time of 30 min. A series of catalysts, K_2_PdCl_4_–Cu(NO_3_)_2_–KBr/S (S = 0.25H-CLI, 0.5H-CLI, 1H-CLI, and 3H-CLI) was obtained. All samples were investigated by X-ray phase and thermogravimetric analysis, FT-IR spectroscopy, water vapor ad/desorption and pH metric method. Besides, K_2_PdCl_4_–Cu(NO_3_)_2_–KBr/S samples were tested in the reaction of low-temperature carbon monoxide oxidation. It have been found that, owing to special physicochemical and structural-adsorption properties of 3H-CLI, it promotes formation of the palladium–copper catalyst providing carbon monoxide oxidation at the steady-state mode down to CO concentrations lower than its maximum permissible concentration at air relative humidity varied within a wide range.

## Backgound

Natural clinoptilolite is a material most commonly used for both water vapor and gaseous toxicant adsorption, gas separation, wastewater treatment. It is also used as an acid catalyst in oil processing and a support for catalytically active phase in the case of catalysts for redox reactions of CO, SO_2_, and O_3_ [1–6]. Catalytic activity of clinoptilolite supported palladium–copper complexes has been found to depend considerably on physicochemical properties and structural parameters of a support affecting a composition of these surface complexes [4, 5]. For optimizing clinoptilolite behavior, one can modify it thermally as well as by treatment with water, acid or alkali at both room and higher temperatures. An effectiveness of the mostly used acid–thermal treatment depends on the nature and concentration of acid applied, a period of interaction between the acid and clinoptilolite (a contact time), and a solid:liquid ratio [7–16]. The acid–thermal modification of clinoptilolite results in a substantial increase in both a Si:Al ratios and its surface acidity [1]. There are also changes in adsorption capacity towards metal ions [17, 18] and water vapor [12, 19], in thermochemical properties [10], in relative crystallinity [13], and in sizes of crystallites [8, 12], and also in structural-adsorption parameters such as a specific surface area (S_sp_), sizes and volumes of pores [7–15].

Properties of acid-modified clinoptilolites of different origin were investigated in many works whereas catalysts composed of clinoptilolite and anchored d metal ions or salts and used for catalyzing redox processes are objects of only few studies. For instance, Ni^2+^/CLI is applied for sulphur removal from fuel oil [18], Ag^+^/CLI [20], Cu^2+^(Zn^2+^, Mn^2+^)/CLI [21], Mn^2+^(Co^2+^, Cu^2+^)/CLI [22, 23] are used for ozone decomposition, K_2_PdCl_4_–Cu(NO_3_)_2_–KBr/H-CLI and CuCl_2_/CLI are proposed by us for the oxidation of carbon monoxide [4–6, 24] and sulfur dioxide [25], respectively.

Although natural zeolites, including clinoptilolite, are commonly used for water vapor adsorption [26–28], adsorption of water vapor by clinoptilolite modified with acid [1, 12, 26] or transition metal ions (complexes) [6, 29, 30] is little-studied. However, it has been found by us [31, 32] that a composition and catalytic performance of surface palladium–copper complexes in some redox processes, namely, carbon monoxide and phosphine oxidation, significantly depend on a thermodynamic activity of water adsorbed on them ($a_{{{\text{H}}_{2} {\text{O}}}}$ = P/P_S_). This parameter was determined from isotherms of water vapor adsorption and proved to be necessary for both obtaining catalysts of optimal composition and their applying in respiratory and environment protection.

Mostly, for clinoptilolite modification, hydrochloric or sulfuric acid [7–14] and, more rarely, phosphoric [33] or nitric [3, 4, 15] acid are used. Our choice of nitric acid as a modifying agent is caused by the following circumstance. Adsorbability of ions in the case of clinoptilolite decreases in the order Cl^−^≫SO_4_^2−^>NO_3_^−^ [34], so, some amounts of chloride and sulfate ions can remain after their desorption by water and, consequently, these residual chloride and sulfate ions, becoming ligands, can decrease the activity of supported palladium–copper complexes [31, 32].

As a rule, acid treatment is used for changing physicochemical and structural-adsorption properties of clinoptilolite. Depending on the aim of a research, acid concentrations may be varied in a wide range [7, 12, 16]. To prepare anchored palladium–copper complexes characterizing by the maximum catalytic activity towards carbon monoxide oxidation, it is necessary to choose an acid concentration optimal for each specific support [35, 36].

The aim of the work is to ascertain how nitric acid concentrations used for clinoptilolite modification affect its physicochemical and structural parameters as well as the catalytic activity of modified clinoptilolite anchored palladium–copper complexes in the reaction of low-temperature carbon monoxide oxidation with air oxygen.

## Experimental

In the work, as in our earlier studies [4, 24], natural clinoptilolite, N-CLI, from Sokirnytsia deposit (Trans-Carpathian region, Ukraine) was used. Acid-modified samples were prepared as follows: 50 g of N-CLI with a grain size of 0.5–1.0 mm were boiled in 100 mL of nitric acid solution with concentrations of 0.25, 0.5, 1.0 or 3.0 mol L^−1^ for 30 min. Then, the samples were washed with bidistilled water till a negative reaction for NO_3_^−^ ions. The obtained samples denoted as 0.25H-CLI, 0.5H-CLI, 1H-CLI and 3H-CLI, respectively, after their air-drying at 110 °C till constant weight, were used for preparation of catalysts by the following procedure: 10 g of each support were subject to incipient wetness impregnation with aqueous solution containing certain amounts of K_2_PdCl_4_ Cu(NO_3_)_2_, and KBr. Loose wet samples obtained were aged in Petri dishes at room temperature for 20–24 h, air-dried in an oven at 110 °C till constant weight, and, finally, cooled in a desiccator over concentrated H_2_SO_4_. As a result, the contents of K_2_PdCl_4_, Cu(NO_3_)_2_, and KBr in all catalyst samples were 2.72 × 10^−5^, 5.9 × 10^−5^, and 1.02 × 10^−4^ mol g^−1^, respectively.

X-ray phase analysis of the samples was carried out with the help of a Siemens D500 diffractometer in Cu*K*_α_ radiation (λ = 1.54178 Å) with a secondary beam graphite monochromator. After thorough grinding, the samples were placed into a glass cell (2 × 1 × 0.1 cm^3^). XRD patterns were collected in 2θ region from 3° to 70° with a step size of 0.03° and an accumulation time of 60 s at every point.

FT-IR spectra were recorded by a Perkin Elmer FT-IR spectrometer (the detection region of 400–4000 cm^−1^ and resolution of 4 cm^−1^). A mixture consisting of a material under study (1 mg) and KBr (200 mg) was compressed under pressure of 7 tons cm^−2^ for 30 s.

A thermogravimetric (DTG–DTA) investigation of the samples (0.25 g) was carried out by a Paulik, Paulik and Erdey derivatograph at a heating rate of 10 °C/min in the temperature range from 20 to 1000 °C with an accuracy of ±5%.

Water vapor ad/desorption by samples of natural and modified clinoptilolite was studied in a vacuum setup with a McBain silica-spring balance thermostated at 21 °C. As a preliminary, the samples (1.0–2.0) × 10^−4^ kg were air-dried at 110 °C till constant weight. Their evacuation was carried out by a fore pump and an oil-vapour diffusion pump for several hours. Residual pressure was monitored by a VIT-2M ionization-thermocouple vacuum meter. A first and following water vapour pumpings were realized till a constant weight attainment. A period of equilibrium achievement for these samples was ca. 24 h. The partial pressure of air was measured with an accuracy of ±2.6 Pa by a U-tube mercury manometer. Both a change in the sample weight caused by adsorption and differences in a U-tube mercury manometer level were measured by a KM-6 cathetometer. Its accuracy was ±2%.

To characterize protolytic properties of surface functional groups, 0.2 g of natural clinoptilolite or its acid-modified samples were suspended in 20 mL of bidistilled water and an equilibrium pH value was measured by a pH-340 instrument equipped with an ESL 43–07 glass electrode and an EVL 1M3 chlorsilver electrode at continuous stirring of the suspension at 20 °C. A suspension effect, ∆pH_s_, was estimated using the following equation

1 $$ \Delta {\text{pH}}_{\text{s}} = {\text{ pH}}_{\text{st}} {-}{\text{ pH}}_{0} $$

where pH_0_ and pH_st_ are pH values of a suspension measured in 15 s and after the equilibrium attainment.

A catalytic activity of the samples in the reaction of CO oxidation was tested in a gas flow setup with a fixed-bed glass reactor at 20 °C. A size of the reactor, an approximate size of catalyst grains, d_g_, equal to 0.75 mm and a linear velocity of gas–air mixture (GAM), U, equal to 4.2 cm s^−1^ fit with the requirements to a kinetically controlled reaction.

A GAM with the initial carbon monoxide concentration, ${\text{C}}_{\text{CO}}^{\text{in}}$, of 300 mg m^−3^ was prepared by attenuation of the concentrated (98–99%) CO with air pre-purified by a tandem filter containing active carbon of SKN-K rank and fibrous filtering material of FP type. ${\text{C}}_{\text{CO}}^{\text{in}}$ and a final carbon monoxide concentration, ${\text{C}}_{\text{CO}}^{\text{f}}$, were measured by a 621EKh04 gas analyzer (Ukraine) with a minimum detectable CO concentration of 2 mg m^−3^.

The reaction rate, W, is evaluated by the equation:

2 $$ {\text{W}} = \frac{{{\text{w}}\left( {{\text{C}}_{\text{CO}}^{\text{in}}  - {\text{C}}_{\text{CO}}^{\text{f}} } \right)}}{{{\text{m}}_{\text{c}} }},\;{{\text{mol}} \mathord{\left/ {\vphantom {{\text{mol}} {\left( {{\text{g}}\times{\text{s}}} \right)}}} \right.} {\left( {{\text{g}}{ \times }{\text{s}}} \right)}} $$

where *w* = 1.67 × 10^−2^ is a volume flow rate of the GAM (L/s), ${\text{C}}_{\text{CO}}^{\text{in}}$ and ${\text{C}}_{\text{CO}}^{\text{f}}$ are initial and final CO concentrations (mol/L), respectively, and m_c_ is a weight of the catalyst sample (g).

A reaction rate constant for steady-state portions of kinetic curves is determined by the equation

3 $$ {\text{k}}_{\text{I}} = \frac{1}{{{\tau^{\prime}}}}\ln \frac{{{\text{C}}_{\text{CO}}^{\text{in}} }}{{{\text{C}}_{\text{CO}}^{\text{f}} }} , {\text{s}}^{ - 1}$$

where τ′ is an effective residence time, calculated as a ratio of a catalyst layer height to a linear velocity of the GAM.

An experimental amount of oxidized CO, Q_exp_, is determined based on experimental $\Delta {\text{C}}_{\text{CO}}^{\text{f}}$vs. τ plots. A percentage of CO conversion at the steady-state mode, η_st_, and a stoichiometric coefficient, n, per 1 mol of Pd(II) (a number of full catalytic cycles) are calculated by the equations

4 $$ \eta_{st} = \frac{{\left( {{\text{C}}_{\text{CO}}^{\text{in}} \text{ - }{\text{C}}_{\text{CO}}^{\text{f}} } \right)}}{{{\text{C}}_{\text{CO}}^{\text{in}} }}{ \times }100,{\% }, $$

5 $$ n = {{{\text{Q}}_{ \exp } } \mathord{\left/ {\vphantom {{{\text{Q}}_{ \exp } } {{\text{Q}}_{\text{Pd(II)}} }}} \right.} {{\text{Q}}_{\text{Pd(II)}} }}, $$

where Q_Pd(II)_ is an amount of palladium(II) contained in the sample.

## Results and discussion

### X-ray characterization

**Fig. 1:**
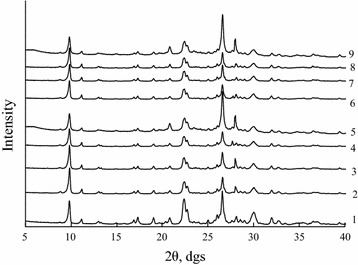
XRD patterns for natural (*1*) and acid-modified 0.25H-CLI (*2*), 0.5H-CLI (*3*), 1H-CLI (*4*), and 3H-CLI (*5*) clinoptilolite samples as well as: Pd(II)–Cu(II)/0.25H-CLI (*6*), Pd(II)–Cu(II)/0.5 H-CLI (*7*), Pd(II)–Cu(II)/1H-CLI (*8*), and Pd(II)–Cu(II)/3H-CLI (*9*) catalysts

Figure 1 shows X-ray diffraction patterns of the samples under study recorded in the 2θ region from 0° to 40° because the most intense reference reflections (2θ (d, Ǻ)) for clinoptilolite phase: 9.865° (8.959), 22.416° (3.963), 30.057° (2.970) and α-SiO_2_ phase: 20.848° (4.257), 26.613° (3.346) are located in this region. The XRD patterns of N-CLI, H-CLI, and Pd(II)–Cu(II)/H-CLI samples were analyzed based on the three reference reflections of the clinoptilolite phase.

X-ray spectral parameters, i.e. an interplanar spacing d (Ǻ), a normalized relative intensity, I_N_, and a relative crystallinity, I_R_ (%) of the samples are summarized in Table 1. I_R_ values were calculated using the procedure described elsewhere [9] as a ratio of the sum of I_N_ values for the three reference reflections taken from XRD patterns of the acid-modified clinoptilolite samples to the sum of those values for N-CLI.

**Table 1:** X-ray spectral parameters for N-CLI, H-CLIs, and Pd(II)–Cu(II)/H-CLIs

Sample	d = 8.955 Å [37]		d = 3.976 Å [37]		d = 2.973 Å [37]		I_R_, %
	d	I_N_	d	I_N_	d	I_N_	
N-CLI	8.959	622	3.963	705	2.970	335	100
0.25H-CLI	8.951	999	3.960	444	2.970	220	100
0.5H-CLI	8.936	999	3.961	481	2.973	229	103
1H-CLI	8.939	999	3.960	538	2.970	260	108
3H-CLI	8.953	608	3.958	553	2.971	240	84
Pd(II)–Cu(II)/0.25H-CLI	8.941	999	3.961	524	2.972	262	107
Pd(II)–Cu(II)/0.5H-CLI	8.953	945	3.962	543	2.974	262	102
Pd(II)–Cu(II)/1H-CLI	8.945	999	3.959	479	2.973	218	94
Pd(II)–Cu(II)/3H-CLI	8.968	378	3.964	285	2.978	122	56

In the case of Pd(II)–Cu(II)/H-CLI samples, I_R_ was determined as a ratio of the sum of I_N_ values for them to the sum of I_N_ values for the corresponding acid-modified clinoptilolite samples. The data presented in Table 1 show that the most significant effect of a nitric acid concentration on I_R_ takes place for ${\text{C}}_{{{\text{HNO}}_{ 3} }}$ = 3.0 mol L^−1^ when the relative crystallinity value goes down to 84% in the case of the 3H-CLI sample and to 56% for the Pd(II)–Cu(II)/3H-CLI one. Deviations observed for the first reference reflection that is usually most sensitive to any structural changes are very slight (0.004–0.017 Ǻ).

Thus, one can deduce that the acid–thermal modification of natural clinoptilolite with nitric acid at its concentration within the range of 0.25 to 3.0 mol/L and the following Pd(II) and Cu(II) anchoring result in some changes in the clinoptilolite structure with no collapse in its framework Moreover, the absence of new X-ray diffraction peaks indicate that no new crystalline phase formed by Pd(II) and Cu(II), i.e. their salts or oxides (PdO, Cu_2_O, CuO) or reduced forms (Pd^0^ or Cu^0^), appears.

### FT-IR characterezation

**Fig. 2:**
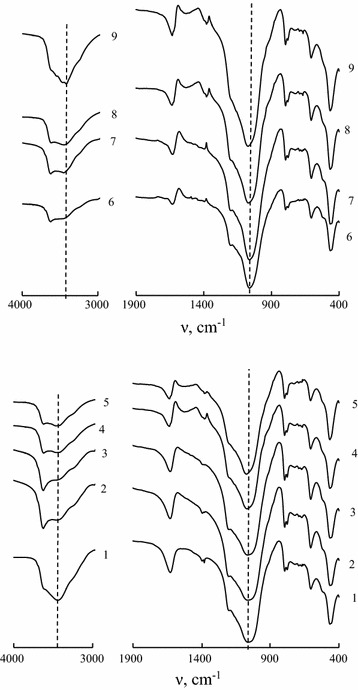
IR spectra for natural (*1*) and acid-modified 0.25H-CLI (*2*), 0.5H-CLI (*3*), 1H-CLI (*4*), and 3H-CLI (*5*) clinoptilolite samples as well as: Pd(II)–Cu(II)/0.25H-CLI (*6*), Pd(II)–Cu(II)/0.5H-CLI (*7*), Pd(II)–Cu(II)/1H-CLI (*8*), and Pd(II)–Cu(II)/3H-CLI (*9*) catalysts

Figure 2 shows portions of FT-IR spectra recorded for N-CLI, H-CLIs, and Pd(II)–Cu(II)/H-CLIs in two regions i.e. 4000–3000 and 1900–400 cm^−1^ because these regions contain the bands characteristic of natural clinoptilolite belonging to the seventh structural group [38]. Results of the FT-IR spectra interpretation are summarized in Table 2.

**Table 2:** Wave numbers (cm^−1^) of absorption band maximums in FT-IR spectra of N-CLI, H-CLIs, and Pd(II)–Cu(II)/H-CLIs

Sample	ν (OH)	δ (OH_2_)	T-O-T (T = Si, Al)			Other bands
			ν_as_	ν_sym_	δ	
N-CLI	3628 sh 3440	1633	1205 sh 1064	797, 778	464	1384, 604
0.25H-CLI	3624 sh 3453	1635	1205 sh 1063	797, 779	464	1399, 607
0.5H-CLI	3624 sh 3454	1635	1205 sh 1068	797, 778	459	1392, 607
1H-CLI	3622 sh 3456	1640	1208 sh 1072	798, 780	467	1531, 1384, 607
3H-CLI	3624 sh 3464	1639	1204 sh 1081	798, 780	467	1531, 1383, 608
Pd(II)–Cu(II)/0.25H-CLI	3621 sh 3446	1638	1209 sh 1064	797, 780	465	1399, 1316, 606
Pd(II)–Cu(II)/0.5H-CLI	3623 sh 3451	1634	1209 sh 1067	797, 780	464	1400, 606
Pd(II)–Cu(II)/1H-CLI	3620 sh 3446	1639	1208 sh 1072	798, 780	467	1537, 1384, 607
Pd(II)–Cu(II)/3H-CLI	3620 sh 3440	1638	1209 sh 1082	798, 780	467	1535, 1384, 607

All FT-IR spectra demonstrate a wide complex-shaped band at ν_OH_ 3440–3484 cm^−1^ which center for 3H-CLI shifts by 24 cm^−1^ in comparison with N-CLI. This band characteristic of stretching vibrations of OH groups in associated water molecules is asymmetrical and its high-frequency component has a clearly detectable shoulder at 3628 cm^−1^ (N-CLI) remainder after the acid treatment and caused by a bridge SiO(H)Al group. Pd(II) and Cu(II) anchoring is accompanied by a low-frequency shift of ν_OH_ indicating a perturbation in hydrogen bonds and a change in their energy induced by metal ions. A band at 1633 cm^−1^ characterizing deformation vibrations of water molecules for N-CLI demonstrates a slight high-frequency shift with the increase in acid concentration, however, it remains unchanged for the samples containing anchored palladium and copper ions (Table 2). A very intense and wide complex-shaped band in the region of 1250–980 cm^−1^ is a superposition of several bands attributed to vibrations of Si–O–Si and Si–O–Al fragments [39].

In the FT-IR spectrum of N-CLI, it is situated at 1064 cm^−1^ and has a shoulder at 1205 cm^−1^. In the FT-IR spectra of the acid-modified samples, the shoulder is in the same position but a center of the band shifts to a high-frequency region and the maximum shift of 17 cm^−1^ is found for 3H-CLI. Pd(II) and Cu(II) anchoring doesn’t change a position of this band in comparison with the corresponding support. For all samples under study, there is no change in positions of the other bands.

The data obtained indicate that, judging from the high-frequency shift of the Si–O–Al band, significant changes in the Si–O–Al structural fragment due to the clinoptilolite dealumination take place after its half-hour acid treatment already at ${\text{C}}_{{{\text{HNO}}_{ 3} }}$ > 0.5 mol L^−1^. Pd(II) and Cu(II) anchoring doesn’t lead to any changes in the frequencies of stretching vibrations of structural groups in the aluminosilicate framework because of low concentrations of these metal ions.

### Thermogravimetric characterization

**Fig. 3:**
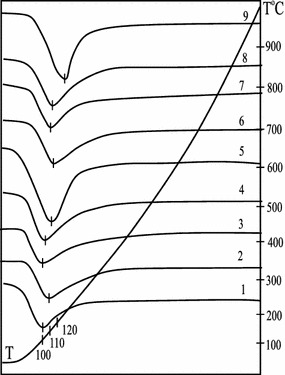
TGA curves for natural (*1*) and acid-modified 0.25H-CLI (*2*), 0.5H-CLI (*3*), 1H-CLI (*4*), and 3H-CLI (*5*) clinoptilolite samples as well as: Pd(II)–Cu(II)/0.25H-CLI (*6*), Pd(II)–Cu(II)/0.5H-CLI (*7*), Pd(II)–Cu(II)/1H-CLI (*8*), and Pd(II)–Cu(II)/3H-CLI (*9*) catalysts

Figure 3 shows differential TGA curves for N-CLI, H-CLI and Pd(II)–Cu(II)/H-CLI samples. Dehydration of the samples is characterized by only one endothermic effect and the temperature corresponding to its maximum coincides with the maximum of its DTG curve. The results of the thermogravimetric analysis are presented in Table 3.

**Table 3:** Results of the thermogravimetric analysis of natural and modified clinoptilolite samples

Sample	T_M_, °C	Weight loss %, in temperature intervals, °C			m$_{{{\text{H}}_{2} {\text{O}}}}$, mmol g^−1^
		25–110	25–300	25–1000	
N-CLI	100	2.4	7.2	12.0	2.7
0.25H-CLI	110	1.6	6.6	10.0	2.8
0.5H-CLI	100	2.2	7.0	10.4	2.7
1H-CLI	100	3.2	8.2	12.0	2.8
3H-CLI	110	2.2	9.2	13.2	3.3
Pd(II)–Cu(II)/0.25H-CLI	110	3.0	8.4	12.4	3.0
Pd(II)–Cu(II)/0.5H-CLI	100	3.0	8.4	13.2	3.0
Pd(II)–Cu(II)/1H-CLI	110	2.8	8.8	12.8	3.3
Pd(II)–Cu(II)/3H-CLI	120	3.6	9.0	12.0	3.1

One can see that the modification of natural clinoptilolite under above mentioned conditions has no substantial influence on T_M_ values. Besides a total weight loss equal to 10–13% for all samples. Weight loss values were estimated for temperature ranges of 25–110 and 25–300 °C in order to quantify specific amounts of water (m $_{{{\text{H}}_{2} {\text{O}}}}$) remained in the samples after their air-drying at 110 °C which are ranged from 2.7 to 3.3 mmol g^−1^.

### Water vapor ad/desorption

**Fig. 4:**
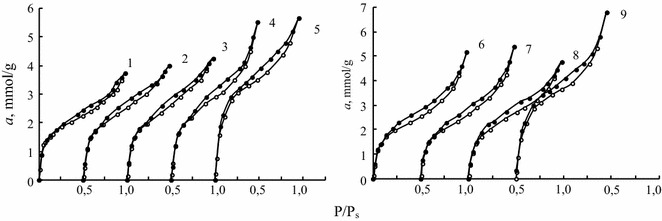
Water vapor adsorption (○) and desorption (●) isotherms for natural (*1*) and acid-modified 0.25H-CLI (*2*), 0.5H-CLI (*3*), 1H-CLI (*4*), and 3H-CLI (*5*) clinoptilolite samples as well as: Pd(II)–Cu(II)/0.25H-CLI (*6*), Pd(II)–Cu(II)/0.5H-CLI (*7*), Pd(II)–Cu(II)/1H-CLI (*8*), and Pd(II)–Cu(II)/3H-CLI (*9*) catalysts at t = 21 °C. Curves 2–5 and 7–9 are shifted one from another by 0.5 P/P_s_

Isotherms of water vapor ad/desorption shown in Fig. 4, are S-shaped and have a clearly defined loops of the capillary condensation hysteresis closed at P/P_s_ < 0.25. Forms of adsorption and desorption branches are similar indicating that the porous structures of the samples don’t change after their exposure to water vapor.

All isotherms obtained by us were analyzed using a linear form of BET equation realized up to P/P_s_ ≤ 0.3 with correlation coefficient R^2^ of 0.98–0.99. A monolayer capacity, *a*_m_, a constant characterizing an affinity between given adsorbate and adsorbent, C, and a specific surface area of the samples, S_sp_, estimated according the procedure described elsewhere [19], are presented in Table 4. Values of a thermodynamic activity of adsorbed water, $a_{{{\text{H}}_{2} {\text{O}}}}$, were determined from the adsorption isotherms shown in Fig. 4 at adsorption values, *a*, equal to the monolayer capacities.

**Table 4:** Structural-adsorption parameters of natural and modified clinoptilolite samples

Sample	Constants of BET equation		S_sp_, m^2^ g^−1^	$a_{{{\text{H}}_{2} {\text{O}}}}$
	*a*_m_, mmol/g	C		
N-CLI	1.53	133.40	100	0.13
0.25H-CLI	1.72	26.3	110	0.13
0.5H-CLI	1.72	26.3	112	0.13
1H-CLI	1.89	29.1	123	0.13
3H-CLI	2.04	10.5	133	0.09
Pd(II)–Cu(II)/0.25H-CLI	1.70	29.9	110	0.13
Pd(II)–Cu(II)/0.5H-CLI	1.70	31.7	110	0.13
Pd(II)–Cu(II)/1H-CLI	1.78	25.4	115	0.13
Pd(II)–Cu(II)/3H-CLI	2.06	10.4	134	0.10

The data presented in Table 4 show that, in comparison with the parameters obtained for N-CLI, values of *a*_m_ and S_sp_ increase and C values diminish with ${\text{C}}_{{{\text{HNO}}_{ 3} }}$ increasing from 0.25 to 3.0 mol L^−1^ in the case of the acid-modified samples. From literature [6–16], it can be seen that a specific surface for acid-modified clinoptilolite significantly depends on an acid concentration, time and multiplicity of treatments, and a solid:liquid ratio. As a rule, S_sp_ increased or attained its maximum value with an acid concentration and only once (S_sp_ determination based on water vapor adsorption) [12], it decreased from 383 to 273 m^2^ g^−1^ when C_HCl_ was heightened from 0.16 to 5.0 mol L^−1^ at the temperature of 100 °C.

In comparison with N-CLI, the $a_{{{\text{H}}_{2} {\text{O}}}}$ value markedly diminishes only for 3H–CLI. The anchored palladium–copper complexes don’t affect the structural-adsorption parameters of the corresponding acid-modified clinoptilolite samples and their water activity values owing to low concentrations of these metal ions.

### pH_s_ characterization

Acid modification of clinoptilolite leads to a drastic change in its protolytic properties that can be quantified by measuring pH of its aqueous suspension. Table 5 summarizes these pH values for N-CLI and H-CLI samples.

**Table 5:** PH values for suspensions of natural and acid-modified clinoptilolite samples

Sample	pH_0_	pH_st_	ΔpH_s_
N-CLI	7.45	8.05	0.6
0.25H-CLI	5.78	5.57	−0.21
0.5H-CLI	5.60	5.35	−0.25
1H-CLI	5.42	5.18	−0.24
3H-CLI	4.38	4.13	−0.25

A directional change in pH values indicates a type of aprotonic sites. For natural clinoptilolite, ΔpH_s_ > 0 showing a prevalence of Lewis basic sites, whereas for acid-modified clinoptilolite forms, ΔpH_s_ < 0, being evidence of a prevalence of Lewis acid sites. Already in the case of 0.25 HNO_3_, pH_st_ lowers from 8.05 to 5.57. A further appreciable decrease in pH_st_ is observed only for 3H-CLI at approximately the same ΔpH_s_ value. Taking into consideration the results of our earlier works [4, 24, 31, 32, 35, 36], this decrease in pH of the aqueous suspension may be one of factors promoting formation of the surface palladium–copper composition optimal for realizing catalytic CO oxidation.

### Testing Pd(II)–Cu(II)/H-CLI samples as catalysts of the reaction of CO oxidation

**Fig. 5:**
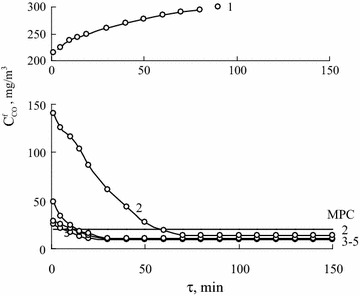
Time dependence of ${\text{C}}_{\text{CO}}^{\text{f}}$ in the course of CO oxidation with air oxygen in the presence of К_2_PdCl_4_–Cu(NO_3_)_2_–KBr/H–CLI sample at different HNO_3_ concentrations of 0 (*1*), 0.25 (*2*), 0.5 (*3*), 1.0 (*4*), and 3.0 (*5*) used for N-CLI treatment C_Pd(II)_ = 2.72 × 10^−5^, C_Cu(II)_ = 5.9 × 10^−5^, C_KBr_ = 1.02 × 10^−4^ mol g^−1^, ${\text{C}}_{\text{CO}}^{\text{in}}$ = 300 mg m^−3^, U = 4.2 cm s^−1^

Kinetic curves in a ${\text{C}}_{\text{CO}}^{\text{f}}$—τ plot obtained as a result of Pd(II)–Cu(II)/H-CLIs testing in the reaction of CO oxidation are shown in Fig. 5. Kinetic and stoichiometric parameters of the reaction in the presence of Pd(II)–Cu(II)/H-CLI catalysts are summarized in Table 6.

**Table 6:** Kinetic and stoichiometric parameters of the reaction of CO oxidation in the presence of K_2_PdCl_4_–Cu(NO_3_)_2_–KBr/S catalysts (S is N-CLI or H-CLIs)

Support	W × 10^9^, mol/(g × s)		${\text{C}}_{\text{CO}}^{\text{f}}$, mg m^−3^	η_st_, %	Q_exp_ × 10^4^, moles of CO	n
	W_in_	W_st_				
N-CLI	4.50	–	300	0	0.78	0.3
0.25H-CLI	10.44	17.16	14	95	13.9	5.1
0.5H-CLI	15.96	17.34	11	96	14.8	5.4
1H-CLI	16.50	17.40	10	97	14.9	5.5
3H-CLI	16.67	17.40	10	97	14.9	5.5

C_Pd(II)_ = 2.72 × 10^−5^, C_Cu (II)_ = 5.9 × 10^−5^, C_KBr_ = 1.02 × 10^−4^ mol g^−1^, ${\text{C}}_{\text{CO}}^{\text{in}}$ = 300 mg m^−3^, U = 4.2 cm s^−1^, d_g_ = 0.75 mm

It should be noted that K_2_PdCl_4_–Cu(NO_3_)_2_–KBr/N-CLI has a very slight activity at the first minute of the GAM feeding, then, final CO concentrations become even greater and equalize to the initial one in 100 min. All other samples permit CO oxidizing at the steady-state mode down to CO concentrations lower than its maximum permissible concentration, MPC_CO_, equal to 20 mg m^−3^ for the working area (Ukrainian Standard). With the increase in ${\text{C}}_{{{\text{HNO}}_{ 3} }}$, some changes in the kinetics of the initial reaction period relating to the formation of catalytically active sites are observed: the time necessary for the steady-state behavior attainment is shortened, ${\text{C}}_{\text{CO}}^{\text{f}}$ values at the steady-state mode are lowered, and initial reaction rate (W_in_) values measured in 5 min of the GAM feeding are heightened. Obviously, the best kinetic parameters are demonstrated by K_2_PdCl_4_–Cu(NO_3_)_2_–KBr/3H-CLI.

**Fig. 6:**
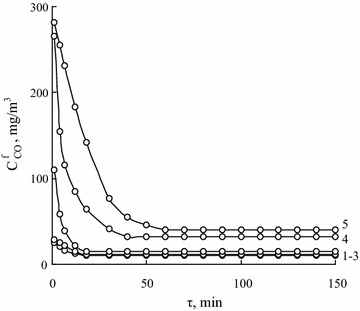
Time dependence of ${\text{C}}_{\text{CO}}^{\text{f}}$ in the course of CO oxidation with air oxygen at different thermodynamic activities of water contained in К_2_PdCl_4_–Cu(NO_3_)_2_–KBr/3H–CLI samples $a_{{{\text{H}}_{2} {\text{O}}}}$: 0.26 (*1*), 0.79 (*2*), 0.87 (*3*), 0.93 (*4*), 1.0 (*5*). C_Pd(II)_ = 2.72 × 10^−5^, C_Cu(II)_ = 5.9 × 10^−5^, C_KBr_ = 1.02 × 10^−4^ mol g^−1^, ${\text{C}}_{\text{CO}}^{\text{in}}$ = 300 mg m^−3^, U = 4.2 cm s^−1^

A thermodynamic activity of adsorbed water depended on both the nature of a support and a composition of an active component anchored on it considerably affect kinetic and stoichiometric parameters of the catalytic carbon monoxide oxidation [32]. A thermodynamic activity of water as a component of K_2_PdCl_4_–Cu(NO_3_)_2_–KBr–H_2_O/3H-CLI catalyst was varied by changing in its content. For this purpose, catalyst samples air-dried at 110 °C till constant weight and containing 3.1 mmol g^−1^ of water (Table 3) were hold in desiccators over 30–35% H_2_SO_4_ solution for 1, 2, 3 or 4 h, As a result of this holding, the contents of additional water in these samples were 1.66, 2.77, 3.32 or 4.44 mmol g^−1^, respectively. Water activity values for each sample were determined from the water vapor isotherms (Fig. 4, curve 9) at the total water contents, Σm $_{{{\text{H}}_{2} {\text{O}}}}$ (Table 7). Figure 6 shows how the thermodynamic activity of water contained in the K_2_PdCl_4_–Cu(NO_3_)_2_–KBr–H_2_O/3H-CLI sample affects its activity in the reaction of CO oxidation. Kinetic and stoichiometric parameters of the reaction in the presence of these catalyst samples presented in Table 7 indicate that the increase in $a_{{{\text{H}}_{2} {\text{O}}}}$ values from 0.26 to 0.87 is accompanied by a very slight decrease (only 2%) in CO conversion values and ${\text{C}}_{\text{CO}}^{\text{f}}$ values remain under MPC_CO_.

**Table 7:** Kinetic and stoichiometric parameters of the reaction of CO oxidation with air oxygen in the presence of K_2_PdCl_4_–Cu(NO_3_)_2_–KBr–H_2_O/3H-CLI samples at different contents of adsorbed water (thermodynamic activities of water)

m $_{{{\text{H}}_{2} {\text{O}}}}$, mmol g^−1^	Σm $_{{{\text{H}}_{2} {\text{O}}}}$, mmol g^−1^	$a_{{{\text{H}}_{2} {\text{O}}}}$	W × 10^9^, mol/(g × s)		${\text{C}}_{\text{CO}}^{\text{f}}$, mg m^−3^	η_st_, %	Q_exp_ × 10^4^, moles of CO	n
			W_in_	W_st_				
0	3.1	0.26	16.7	17.4	10	97	14.9	5.5
1.66	4.77	0.79	16.5	17.3	12	96	14.8	5.4
2.77	5.87	0.87	14.5	17.1	15	95	14.6	5.4
3.32	6.42	0.93	8.8	16.1	32	89	13.4	4.9
4.44	7.54	1.0	2.7	15.6	40	87	12.3	4.5

C_Pd(II)_ = 2.72 × 10^−5^, C_Cu (II)_ = 5.9 × 10^−5^, C_KBr_ = 1.02 × 10^−4^ mol g^−1^, ${\text{C}}_{\text{CO}}^{\text{in}}$ = 300 mg m^−3^, U = 4.2 cm s^−1^, d_g_ = 0.75 mm

At $a_{{{\text{H}}_{2} {\text{O}}}}$ close to 1.00, the catalyst loses its protective properties

As in the case of ${\text{C}}_{{{\text{HNO}}_{ 3} }}$ varying (Fig. 5), the $a_{{{\text{H}}_{2} {\text{O}}}}$ varying causes the most appreciable changes in the kinetics of the initial reaction period relating to the formation of catalytically active palladium–copper complexes. Besides the steady-state mode of the reaction proceeding, the catalytic nature of the process is confirmed by the fact that the stoichiometric coefficients of the reaction, n, are more than 1 (Tables 6, 7) indicating a multiple participation of palladium(II) in the process.

Thus, the best catalytic behavior in the reaction of CO oxidation is demonstrated by the palladium–copper catalyst based on acid-modified clinoptilolite obtained as a result of half-hour boiling in 3 M HNO_3_.

## Conclusions

Acid modified forms of clinoptilolite prepared by acid–thermal treatment of natural clinoptilolite with 0.25, 0.5, 1.0, and 3 M HNO_3_ were used as supports for a palladium–copper composition to obtain samples catalytically active in the reaction of carbon monoxide oxidation. The comparative study of natural and chemically modified samples was performed using XRD and FTIR spectroscopic methods, pH-metry, thermogravimetric analysis, and water vapor adsorption. Spectroscopic methods demonstrated that the maximum degree of natural clinoptilolite dealumination without damage of aluminosilicate framework was attained in the case of 3 M HNO_3_ treatment (3H-CLI). pH-metry showed that the highest surface acidity was achieved also in the case of 3H-CLI, promoting formation of active anchored palladium–copper complexes. The isotherms of water vapor ad/desorption suggested that the highest specific surface area was, again, obtained for 3H-CLI and K_2_PdCl_4_–Cu(NO_3_)_2_–KBr-H_2_O/3H-CLI samples. It should be noted that there was no change in XRD, FTIR, and pH-metry parameters after anchoring Pd(II) and Cu(II) on 3H-CLI. Also, thermogravimetric analysis demonstrated that the residual water adsorption value in K_2_PdCl_4_–Cu(NO_3_)_2_–KBr–H_2_O/3H-CLI after its air-drying at 110 °C (the temperature used in our procedure of catalyst preparation) was 3.1 mmol g^−1^, and this value, according to the water vapor isotherm, corresponded to $a_{{{\text{H}}_{2} {\text{O}}}}$ = 0.26 (i.e. relative humidity, RH, of 26%). Notably, this catalyst composition was the most active in CO oxidation. Testing CO oxidation at increasing RH showed that the catalyst retained almost all of its activity at RH increasing up to 87%. Thus, this catalytic composition can purify air from carbon monoxide in a steady-state mode down to MPC_CO_ at a wide range of RH and, therefore, can be used in respirators protecting against CO.
